# Animal models for COVID-19: advances, gaps and perspectives

**DOI:** 10.1038/s41392-022-01087-8

**Published:** 2022-07-07

**Authors:** Changfa Fan, Yong Wu, Xiong Rui, Yuansong Yang, Chen Ling, Susu Liu, Shunan Liu, Youchun Wang

**Affiliations:** 1 Division of Animal Model Research, Institute for Laboratory Animal Resources, National Institutes for Food and Drug Control (NIFDC), National Rodent Laboratory Animal Resources Center, Beijing, 102629 China; 2 Department of Microbiology & Infectious Disease Center, School of Basic Medical Sciences, Peking University Health Science Center, Beijing, 100083 China; 3 College of Life Sciences, Northwest University; Provincial Key Laboratory of Biotechnology of Shaanxi Province, Northwest University, Xi’an, 710069 China; 4 Division of HIV/AIDS and Sexually Transmitted Virus Vaccines, Institute for Biological Product Control, National Institutes for Food and Drug Control (NIFDC), Beijing, China

**Keywords:** Experimental organisms, Vaccines

## Abstract

COVID-19, caused by SARS-CoV-2, is the most consequential pandemic of this century. Since the outbreak in late 2019, animal models have been playing crucial roles in aiding the rapid development of vaccines/drugs for prevention and therapy, as well as understanding the pathogenesis of SARS-CoV-2 infection and immune responses of hosts. However, the current animal models have some deficits and there is an urgent need for novel models to evaluate the virulence of variants of concerns (VOC), antibody-dependent enhancement (ADE), and various comorbidities of COVID-19. This review summarizes the clinical features of COVID-19 in different populations, and the characteristics of the major animal models of SARS-CoV-2, including those naturally susceptible animals, such as non-human primates, Syrian hamster, ferret, minks, poultry, livestock, and mouse models sensitized by genetically modified, AAV/adenoviral transduced, mouse-adapted strain of SARS-CoV-2, and by engraftment of human tissues or cells. Since understanding the host receptors and proteases is essential for designing advanced genetically modified animal models, successful studies on receptors and proteases are also reviewed. Several improved alternatives for future mouse models are proposed, including the reselection of alternative receptor genes or multiple gene combinations, the use of transgenic or knock-in method, and different strains for establishing the next generation of genetically modified mice.

## Introduction

At the time of writing, the fourth world-wide pandemic declared by WHO on 11 March 2020,^1,2^ which was caused by a novel coronavirus identified firstly in December 2019,^2–5^ is still going on in the spring of the third year. The contagious disease caused by severe acute respiratory syndrome coronavirus 2 (SARS-CoV-2) was termed officially as coronavirus disease 2019 (COVID-19) on 11 February 2020 by the WHO.^6–8^ Until now, cumulative confirmed cases exceeded 400 million, and over 5.8 million deaths have been reported globally.

In particular, the highly contagious Omicron variant (B.1.1.529) infected 84 million people in the first month of 2022 alone, equivalent to the number of infections for the whole of 2020 (https://www.who.int/emergencies/diseases/novel-coronavirus-2019/situation-reports).^9,10^ Two previous outbreaks of highly contagious coronaviruses in humans before COVID-19 were caused by severe acute respiratory syndrome coronavirus (SARS-CoV) in 2002–2003,^11,12^ and the Middle East respiratory syndrome coronavirus (MERS-CoV) in 2012.^13,14^ The transmission of SARS-CoV-2 among humans, which is similar to the previous two coronaviruses but more difficult to control, occurs through direct contact, respiratory droplets, contaminated objects, and aerosols.^4,15–20^ The long-term and large-scale epidemic of SARS-CoV-2 characterized by widespread community transmission,^21^ while causing large numbers of asymptomatic and mild cases,^22,23^ and was also transmitted from humans to animals^24,25^ has brought great pressure on the public health of all countries in the world.

Six coronaviruses that infect humans generally belong to one of two categories. One includes common human coronaviruses known to infect immunocompromised individuals,^5^ including HCoV-229E (alpha coronavirus), HCoV-NL63 (alpha coronavirus), HCoV-OC43 (beta coronavirus), and HKU1 (beta coronavirus). The other category includes zoonotic species from the Beta coronavirus genus, including MERS-CoV and SARS-CoV.^26,27^ SARS-CoV-2 is considered the seventh member of the coronavirus family to successfully infect humans (Coronaviridae Study Group of the International Committee on Taxonomy of Viruses 2020).

SARS-CoV-2 has a virion morphology similar to other coronaviruses. Electron micrographs revealed that SARS-CoV-2 virions were roughly spherical with some pleomorphism, ranging in size from 60 to 140 nm in diameter.^28^ It is an enveloped virus with a positive‐sense single‐stranded RNA genome. The genome sequence of SARS‐CoV‐2 was found to be 79.4% similar with SARS-CoV and 50% identical with MERS-CoV. SARS-CoV-RaTG13 (a bat CoV) was found to share 96% identity with SARS‐CoV‐2.^28,29^ Genome sequences confirmed the virus as CoV and indicated a common ancestor based on the high similarity between bat CoV and SARS-CoV-2.

Due to the high sequence similarity between SARS-CoV-2 and SARS-CoV-1, their ORFs and nonstructural proteins (nsps) are not significantly different.^30^ The whole genome of SARS-CoV-2 is ~29.8 kb in length with several open-reading frames (ORFs), the number of which varies across the CoVs.^31^ The first ORF (ORF1a/b), encoding polyproteins required for viral replication and transcription (nsp1-nsp 16), occupies two-thirds of the viral genome, while the remaining 13 ORFs encode accessory and structural proteins,^32^ including spike (S), membrane (M), envelope (E), and nucleocapsid (N).^33–35^ Particularly, the receptor-binding domain (RBD) of the spike protein (S) is the primary target of neutralizing antibodies,^36^ and mutations in S and its RBD largely determine the effectiveness of SARS-CoV-2^37^ vaccines and the transmissibility of SARS-CoV-2.^38^ By 2022, many variants of concern (VOC) have emerged, accumulating multiple mutations mainly in the spike gene.^39^ These mutations can enhance the infectivity and virulence of SARS-CoV-2, lead to changes in clinical disease manifestations, or affect the effectiveness of diagnostics, vaccines, and treatment strategies.^40^

Since the outbreak of the pandemic, considerable efforts have been made to develop effective and safe vaccines, and therapeutic drugs, as well as to understand etiopathogenesis and immunology of SARS-CoV-2 infection. Animal models play crucial roles in all these studies. In the beginning of 2020, the WHO COVID Modeling group (WHO-COM), whose goal was to develop animal models of SARS-CoV-2 infection as soon as possible, was convened quickly by the World Health Organization (WHO) R&D Blueprint.^41,42^ Various animals were employed to develop COVID-19 disease models, including non-human primates (NHPs), genetically modified mice, wild-type mice sensitized by Ad5 or AAV vectors expressing the hACE2 gene, as well as Syrian hamster, ferret, poultry, and domestic animal models. Here, we summarized the success and deficits of current animal models, as well as the classical and alternative SARS-CoV-2 receptors and proteases. Then, the gaps in current animal models are analyzed and strategies for the development of further COVID-19 animal models are proposed, we strongly believe that it is necessary to develop and reserve animal resources in advance, as well as establish a rapid breeding supply system to be ready for the next pandemic.

## Clinical features of COVID-19

### General clinical symptoms

**Fig. 1:**
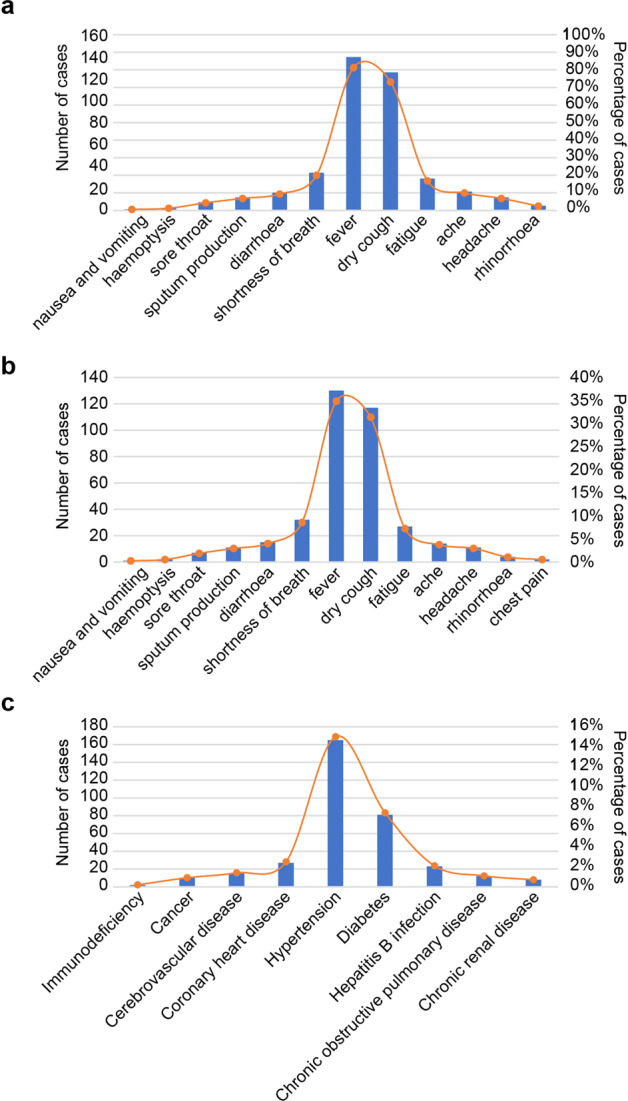
The percentage of clinical symptoms of COVID-19 patients with and without comorbid disease in all cases. **a** The symptoms of patients with confirmed SARS-CoV-2. **b** The most common signs of COVID-19 patients with comorbid disease. **c** Comorbid disease may aggravate COVID-19. The denominator of percentage in this figure is total cases. This represents the percentage of all cases

Based on the clinical observation of 1198 patients with laboratory-confirmed COIVD-19 from more than 600 hospitals, the characteristic presentation of SARS-CoV-2 infected patients is pneumonia^43,44^ accompanied by other related symptoms.^35,45–48^ The most common symptoms were fever (more than 90%) and cough (more than 80%),^49,50^ while rhinorrhea and nausea were uncommon.^51,52^ In another report, clinical symptoms included fever, cough, shortness of breath, fatigue, muscle ache, diarrhea, headache, increased sputum production, sore throat, rhinorrhea, hemoptysis, nausea, and vomiting.^26,53–55^ The clinical symptoms and incidence were summarized in Fig. 1a.

Variants of concern cause diverse clinical symptoms and have varying incidence.^56,57^ The alpha VOC was found to result in 3.8-fold higher risk of death or transfer to the ICU compared to the original strain,^58,59^ while also being characterized by more common loss of smell or taste.^59^ By the end of 2021, the case fatality rate was ~3% worldwide. Based on a large sample from the general population, without declaring their past medical history. However, there are differences in morbidity and mortality in specific subpopulations, such as pregnant women, babies, or patients with underlying medical conditions, such as diabetes, cancer, or cardiovascular disease.^60^ According to a report by the Chinese Centre for Disease Control, based on 44,672 patients, the case fatality rate was 7.3% in patients with diabetes as opposed to 2.3% in ono-diabetics.^61^ In addition, some studies have reported cases with atypical symptoms. For example, Li et al. reported conjunctivitis as the first symptom in two patients,^61^ while Yesilkaya et al. reported psychosis in two cases.^62^

### Infection in different populations

Everyone is generally susceptible to SARS-CoV-2,^63,64^ but there are differences in the symptoms of infection among patients of different ages.^65^ Approximately 80% of older adults aged over 65 years with severe COVID-19 infection are admitted to the ICU, while there are few ICU admissions in patients younger than 19.^66,67^ However, once children are severely infected, treatment is not easy, medication is limited, and the prognosis is unknown, which should also be paid more attention.^68^

Older adults, especially those with underlying chronic diseases are more likely to develop severe or critical illness,^69,70^ resulting in hospitalization, acute respiratory distress syndrome, and death.^46,71^ Deaths are more common in the elderly over the age of 65 (more than 70%).^72^

Elderly patients also often have fever (almost 80%) and cough (almost 50%) as the main symptoms,^73^ followed by dyspnea, asthenia, anorexia, chest tightness, diarrhea, and to a lesser extent myalgia, pharyngitis, nausea, dizziness, headache, abdominal pain, vomiting and other symptoms.^35,43,72,74–76^ In older adults, which are often susceptible to underlying diseases, are also more susceptible to SRAS-CoV-2, which needs special attention.^5,77^

Pregnant patients with SARS-CoV-2 infection showed fever (7/9), cough (4/9), myalgia (3/9), sore throat (2/9), diarrhea (1/9), and dyspnea (1/9). Fortunately, there were no signs of coronavirus infection in their newborn babies, indicating that there is no evidence of vertical transmission.^78,79^ In another study, 23 pregnant women were investigated, and all 25 neonates were born alive. Three of the neonates were transiently suspected positive for SARS-CoV-2 after birth, but no newborns developed COVID-19.^80^

Children represent ~15% of the total number of COVID-19 cases.^61^ Although the clinical course of COVID-19 is relatively mild in children,^81^ we cannot ignore it, as the increasing number of cases in children may result in a higher number of patients.^68^ Children were mainly infected within the family, usually showing typical symptoms of acute respiratory infection,^44^ including fever (generally below 39 °C) and cough (more than 50%),^43,61^ Different from adults, the clinical picture of COVID-19 in children includes skin lesions as well as respiratory, gastrointestinal, and also neurological symptoms.^82^ Numerous reports describe significant abnormalities in a relatively small proportion of children infected with SARS-CoV-2.^83,84^ In newborns, the screening begins with primary maternal infection (84%). Similarly, most older children were asymptomatic (20%) or had mild (48%) and moderate (20%) signs of clinical infection.^85,86^ However, a slightly higher proportion of older children became severely ill (12%).^87^ Dyspnea was the most commonly reported sign in neonates (40%).^62,80^

The sex disparities in COVID-19 severity and outcome have been described.^88^ It seems that males are more susceptible to COVID-19 than females presumably because of smoking,^89,90^ a less effective immune response, and a greater predisposition to thromboembolism, ultimately resulting in severe clinical manifestations in males. In addition, the viral load of the Delta variant was found to be higher in female than in male patients, but that of other variants was almost equal.^91^

### Infection in patients with preexisting diseases

A study of 41 patients, included 32% of patients with underlying chronic conditions such as cardiovascular, diabetes, or hypertension. Similar to the general population without preexisting disease, the chronic patients showed clinical symptoms such as fever, cough, fatigue, increased sputum production, headache, hemoptysis, and diarrhea (Fig. 1b),^43^ but they had higher rates of fever and fatigue,^92^ which may be the result of an impaired immune response.^46^

Patients with comorbidities account for ~30% of all patients with COVID-19, with conditions such as chronic obstructive pulmonary disease, diabetes, hypertension, cardiovascular disease, cerebrovascular disease, hepatitis B infection, cancer, chronic renal disease, and immunodeficiency.^46,51,93–96^ The incidence of these comorbidities is shown in Fig. 1c. Diabetes, hypertension, and cardiovascular and cerebrovascular diseases are the most common preexisting disease among COVID-19 patients.^15,35,36,43^

Diabetes is an important risk factor for adverse outcomes in COVID-19. In addition, cancer patients infected with SARS-CoV-2 have a poor prognosis, especially in case of hematological malignancy, with the state of illness being more evident and resulting in a higher risk of death.^97,98^ Male sex and aging are high‑risk factors for COVID‑19 patients with cancer.^84,99^ Patients with two or more comorbidities had significantly escalated risks compared with those who had single comorbidity, while patients with chronic obstructive pulmonary disease, diabetes, hypertension, and malignancy were more likely to reach the composite endpoints than those without.^94,100^

### Infections of the central nervous system

Coronaviruses not only cause respiratory illness but can also invade the central nervous system through a synapse-connected route, which was observed in both patients and the brains of experimental animals. Some early COVID‐19 patients presented neurological signs such as nausea, headache, and vomiting, with fewer neurological symptoms, but with the increase of cases and the emergence of VOC, more cases showed neurological disease symptoms.^62,101,102^ More than 70% of patients presented related physical symptoms, lasting between 1 and 35 days. The most common reported symptom of psychosis was delusions, hallucinations disorganized thought, behavior, and speech, or even catatonia.^103^

Anosmia is a common and often the sole symptom of COVID-19 patients,^55^ and numerous clinicians around the world have reported smell/taste impairment as a symptom of the disease.^76^ Studies have found that more than 80% of patients have a decreased sense of smell and taste, whereby more than 60% of them have completely lost the sense of smell and taste after general symptoms.^104^ Notably, studies indicate that the olfactory sensory neurons are affected without getting infected.

### Infection and injury of the gastrointestinal tract

In addition to invading the respiratory system, SARS-COV-2 also invades other organs expressing the cell surface receptor ACE2, and the digestive system is a susceptible target of SARS-COV-2.^105,106^ More than 80% of COVID-19 patients have one or more digestive symptoms along with fever over the course of the disease.^107^ In some patients, this was the first, or only, a symptom of the disease.^108,109^ It was found that COVID-19 patients with gastrointestinal symptoms mostly developed the severe disease, resulting in a high risk of death.^110,111^ Accordingly, gastrointestinal symptoms may be an important prognostic factor in COVID-19,^112,113^ and it is valuable to establish an intragastric challenge mouse model.^114^

### Hematological and biochemical parameters

Hematological and biochemical parameters are widely used to diagnose infections, especially those caused by viruses.^115,116^ Patients infected with SARS-CoV-2 have changed hematological and biochemical parameters, which may be evidence for the infectious type and severity of the disease.^117,118^ During diagnosis and treatment, hematological indices such as the counts of erythrocytes, leukocytes, lymphocytes, neutrophils, and platelets are monitored. Usually, biomarkers such as IL-6, hemoglobin, serum ferritin, C-reactive protein (CRP), rate of erythrocyte sedimentation, alanine aminotransferase (ALT), aspartate aminotransferase (AST), creatine kinase and D-dimer are tested as well.^75,119,120^

In a sample of more than 800 patients, thrombocytopenia was reported in more than 30% of patients with COVID-19,^117,121,122^ and patients with severe disease had higher levels of thrombocytopenia than those with non-severe disease. Thus, a high platelet count can be used as an indicator of good prognosis.^123^ A study by Guan et al., based on blood samples of 1099 patients with COVID-19 in China showed that more than 80% of patients had lymphopenia, and the ratio of leucocytes was lower than the normal range in 33.7% of infected patients.^121^ Reduced hemoglobin levels have been noted in some severe COVID-19 patients, but 20–40% of patients have leucopenia, 3–24% have leukocytosis, and lymphopenia was seen in 30–75% of COVID-19 patients.^124^

Blood samples collected from 24 asymptomatic carriers with confirmed novel coronavirus infection showed that most of the patients had increased levels of ALT, AST, CPR, D-dimer, and creatine kinase.^101,125^ Moreover, 16.7% of patients had decreased lymphocyte ratios, which was lower than in the report by Guan and colleagues.^121^ A study reports that at least 5% of patients have heart failure.^126^ Among them, the most common cardiac pathology is right ventricular (RV) dilatation (39%), followed by left ventricular (LV) diastolic dysfunction (16%) and left ventricular systolic dysfunction (10%). Although about 60% of patients can recover eventually, patients with clinical heart failure have a high risk of poor prognosis of COVID-19 infection.^127^

Hemophagocytosis has been noted in the bone marrow aspirates of three severe COVID-19 patients, whereby increased numbers of megakaryocytes in the bone marrow were also found.^128,129^ Thrombosis also commonly occurs in patients with COVID-19, including pulmonary embolism, venous thromboembolic events such as proximal deep vein and upper extremity thrombosis, as well as arterial thromboembolic events such as ischemic stroke.^130,131^

### Effects on the reproductive system

In an early epidemiological survey, one patient was reported to develop testicular pain,^132^ and about 40% of patients had low libido, suggesting that SARS-CoV-2 infection may affect the reproductive function of patients. Moreover, it has been reported that erectile failure results from COVID-19-caused cardiovascular dysfunction, and the subsequent treatment may further deteriorate libido.^133–135^

Although the SARS-CoV-2 virus was not found in the testes of COVID-19 patients, testicular microscopy showed spermatocyte abscission and sperm cell elongation, suggesting acute testicular injury.^136^ Consistently, spermatogenic tubule damage and spermatocyte shedding were observed in the testicular cells of patients infected with COVID-19, but no viral particles were detected in testes^137^ and semen.^136,137^

## Receptors and cellular proteases that mediate SARS-CoV-2 infection

Cellular receptors mediate viral attachment and the fusion of the viral envelope with the host cell membrane,^138–140^ leading to infection of the host cell. Endogenous proteases,^141–143^ are also required for viral fusion with the cellular membrane and entry into target cells (Table 1). Several classical cellulars and alternative receptors for SARS-CoV-2 have been reported.^139,144^ The classical receptor human angiotensin-converting enzyme 2 (hACE2)^145^ is used by both for the first SARS-CoV^146–149^ and SARS-CoV-2.^150–152^ Alternative receptors include extracellular matrix metalloproteinase inducer (EMMPRIN or CD147),^153^ asialoglycoprotein receptor 1 (ASGR1),^154^ kringle containing transmembrane protein 1 (KREMEN1),^154^ neuropilin‐1 (Nrp1),^155^ dipeptidyl peptidase 4 (DPP4/CD26),^156^ alanyl aminopeptidase (ANPEP/CD13),^156^ angiotensin II receptor type 2 (AGTR2),^157^ and glutamyl aminopeptidase (ENPEP).^156^

**Table 1:** Possible receptors and proteases for SARS-CoV-2 and other coronavirus entry

Virus	Cellular receptors	Expression profile	In vivo supporting data available or not	References
SARS-COV-2; SARS-CoV-1	Human angiotensin-converting enzyme 2 (ACE2)	High expression in the small intestine, testis, kidneys, heart, and thyroid. Medium expression in the lungs, large intestine, bladder, liver, and adrenal glands	Yes, several mouse modes expressing human ACE2 available, and with lots of challenging experiments	Zhou et al.^175^; Hoffmann et al.^171^
SARS-COV-2	Extracellular matrix metalloproteinase inducer (EMMPRIN/CD147)	High expression in tumor tissues and inflamed tissues	Yes, hCD147Tg-NSG mouse model available and challenged	Badeti et al.^188^
SARS-COV-2	asialoglycoprotein receptor 1 (ASGR1)	RNA expression mainly in liver, protein expression in liver, stomach, gallbladder^a^	Yes, a mouse transduced by lentiviral particles encoding human ASGR1, KREMEN1 or ACE2 available	Gu et al.^154^
SARS-COV-2	Kringle containing transmembrane protein 1 (KREMEN1)	Wildly expressing in all tissues, but higher in esophagus, heart muscle, skeletal muscle and skin^b^	Yes, a mouse transduced by lentiviral particles encoding human ASGR1, KREMEN1 or ACE2 available	Gu et al.^154^
SARS-COV-2	Neuropilin‐1 (Nrp1)	The respiratory epithelium, olfactory epithelium, endothelial cells, excitatory neurons, and nasal cavity epithelial cells	No, no mouse model expressing human Nrp1 available	Cantuti‐Castelvetri et al.^155^
SARS-COV-2 MERS-CoV	Dipeptidyl peptidase 4 (DPP4/CD26)	The kidneys, lungs, smooth muscle, liver, and capillaries	No, mouse models expressing hdPP4 and challenged by MERS-CoV but not for SARS-CoV-2	Raj et al.^198^; Fan et al.^202^
SARS-COV-2	alanyl aminopeptidase (ANPEP/CD13)	High expression in the ileum, colon, rectum, kidneys, skin, and liver	No, a transgenic mouse model expressing porcine aminopeptidase N available and be challenged	Qi et al.^156^
SARS-COV-2	angiotensin II receptor type 2 (AGTR2)	High expression in the lungs	No, no transgenic mouse model expressing human AGTR2 available	Cui et al.^130^
SARS-COV-2	Glutamyl aminopeptidase (ENPEP)	Expression pattern similar to ACE2 expression	No, no transgenic mouse model expressing human AGTR2 available	Qi et al.^156^

Virus	Proteases			
SARS-COV-2 HCoV-OC43	Transmembrane serine protease 2 (TMPRSS2)	Type II pneumocytes, subsegmental bronchial branches, enterocytes in the small intestine, heart, liver, kidney, and neurons	No, no transgenic mouse model expressing human TMPRSS2, but knockout mouse available	Hoffmann et al.^171^
SARS-CoV-2	Cathepsin B and L, a lysosomal proteases	High expression in the heart	No, no transgenic mouse model expressing human Cathepsin B and L available	Hoffmann et al.^171^
SARS-CoV-2	Furin, an endogenous serine protease,	Small intestine	No, mouse models expressing furin available, but not be challenged by SARS-CoV-2	Zhou et al.^221^
SARS-CoV-2	Trypsin, serine endopeptidase;	Respiratory cells and gastrointestinal cells, particularly in the small intestine	No, no in vivo experimental data available	Ou et al.^219^

^a^https://www.proteinatlas.org/ENSG00000141505-ASGR1/tissue

^b^https://www.proteinatlas.org/ENSG00000183762-KREMEN1/tissue

### Classical receptor and novel receptor candidates

#### Angiotensin‐converting enzyme 2

Human ACE2 is the most widely recognized receptor for SARS-CoV-2, which has been supported by numerous in vitro^158–162^ and in *vivo* experiments.^114,144,163–166^ Moreover, it has been identified as a receptor for SARS-COV-1 since 2003.^146,147^ ACE2 was discovered in early studies as vital regulator of molecular in cardiac function and various other organs via the renin–angiotensin system (RAS).^167–169^ The receptor‐binding domain of the viral S protein binds to ACE2,^124,170,171^ causing SARS‐CoV‐2 to undergo endocytosis and exposes it to endosomal proteases leading to viral infection of the host.^172–174^ HeLa cells expressing hACE2 are susceptible to infection with SARS‐CoV‐2.^175^

The main impediment to the infection of wild-type mice with SARS-CoV-2 is lack of appropriate receptors to initiate viral infection.^41^ The mouse *Ace2* gene (m*Ace2*) gene was mapped to chromosome X 70.5 cM. It produces two cDNAs with respective lengths of are 2746 and 1995 bp due to alternative splicing. Notably, mACE2 showed only 83% identity with hACE2,^176^ resulting in a lack of binding by SARS-CoV-2, preluding infection. The successful development of mouse models by transgenic,^165,177^ humanization,^178^ or by Ad5^179^ transduction have proved the function of ACE2 as a necessary and sufficient factor for infection.

Sequencing of normal human tissues^32^ and normal human lung samples,^180^ has demonstrated the expression hACE2 in type II pneumocytes, which appear to support viral replication in humans. However, studies do not indicate that SARS‐CoV‐2 can infect all organs expressing the ACE2 receptor, and other factors, such as cellular proteases that cleave the viral S protein, may also be required.^181^

#### CD147

CD147 is a transmembrane glycoprotein, commonly known as an extracellular matrix metalloproteinase inducer (EMMPRIN) or basic immunoglobulin. In earlier studies, CD147 overexpression was found in most cancers, including melanoma, where it was associated with a poor prognosis^182^ and inflammation.^183^ Accordingly, CD147 was considered a cancer-associated biomarker with potential role in cancer detection.^182^ It is found in brain tissue rather than the respiratory system.^184^

CD147 has been demonstrated to be involved in human immunodeficiency virus (HIV)-1 infection by interacting with virus-associated cyclophilin A.^185^ Chen et al.^186^ reported the role of CD147 in invasion of host cells by SARS-CoV-1 in 2005. Recently, CD147-spike protein interaction was revealed as a novel route for SARS-CoV-2 infection of host cells.^153^ Cells in the central nervous system are more likely to be infected with SARS-CoV-2 through the CD147 receptor and TMPRSS2 protease than through hACE2, since studies revealed that TMPRSS2 and CD147 mRNA levels were higher in the pituitary area, cerebellum, and cortex of the mouse brain.^184^ This result may explain the mechanism of SARS-CoV-2 infection in the central nervous system.^187^

A hCD147Tg-NSG mouse model was successfully generated in the NOD- *scid* IL2Rgamma ^null^ (NSG) background.^188^ In this model, the human CD147 sequence was knocked-in after the endogenous promoter of mouse CD147 (*mCD147*), resulting in physiological expression of human CD147 protein in appropriate mouse tissues. The novel hCD147Tg-NSG mouse model might allow more detailed studies of the pathogenicity of SARS-CoV-2 in immunocompromised patients. In addition, this mouse model can confirm if CD147 serves as an independent functional receptor or accessory receptor for SARS-CoV-2 entry.^188^

#### Asialoglycoprotein receptor 1 and Kringle containing transmembrane protein 1

Asialoglycoprotein receptor 1 (ASGR1) mediates the internalization of galactose-terminated glycoproteins into hepatocytes for degradation in lysosomes. The mouse ASGR genes have been mapped to mouse chromosome 11 using recombinant inbred stains, while the human ASGR 1 and 2 genes, which encode the major Hl and minor H2 receptor polypeptides, are located on chromosome 17p11-13.^189^ ASGR RNA expression is mainly detected in the liver, with protein expression detectable in the liver, stomach and gallbladder. Kremen proteins are Dickkopf receptors regulate Wnt/β-catenin signaling,^76^ and are wildly expressed in different tissues.

Genomic receptor profiling identified ASGR1 and KREMEN1, together with ACE2, as potential receptors with diverse S-binding affinities and patterns,^154^ implying that they may be alternative functional receptors for SARS-CoV-2 cell entry. Cells expressing ACE2/ASGR1/KREMEN1 receptor combinations display a markedly stronger virus susceptibility than those expressing any individual receptor at both the cell and tissue levels. Mouse models transduced with lentiviral particles encoding human ASGR1, KREMEN1 or ACE2 supported SARS-CoV-2 infection. These alternative receptors, independent of ACE2, provide insights into SARS-CoV-2 tropism and pathogenesis, which can inform potential therapeutic strategies.^154^

The physiological expression pattern of ASGR1, which is not expressed in the lungs, trachea, bronchus, brain and other organs that are affected in clinical cases, makes it a less likely alternative receptor supporting SARS-CoV-2 infection. A lentivirus transduced mouse model provided in vivo evidence, but this conjecture needs further confirmation, for example using transgenic mouse model.

#### Neuropilin‐1

The neuropilin (NRP) family consists of two members, NRP1 and NRP2.^190^ As a transmembrane glycoprotein, neuropilin 1 acts as a co-receptor for a number of extracellular ligands, including transforming growth factor beta, class III/IV semaphorins, and certain isoforms of vascular endothelial growth factor.^191^ NRP1 is expressed on a subset of T regulatory cells and in plasmacytoid dendritic cells, where it aids in priming immune responses. In mice, it is selectively expressed on thymic-derived Tregs and greatly enhances their immunosuppressive function. NRP1 is highly expressed in macrophages and DCs but not CD4+ T cells, serving as an anti-HIV factor to inhibit the infectivity of HIV-1 progeny virions.^192^ Down-regulated expression of NRP-1 significantly enhanced the transmission of HIV-1 in macrophages and dendritic cells, while also increasing the infectivity of HIV-1 virions.

Recently, it has been observed that SARS-CoV-2 infection promotes liver injury through pathways that may be influenced by previous pathological status and liver expression of NRP1. The cytokine storm in infected patients with severe disease may influence liver sinusoidal-cell phenotype, and facilitating viral invasion.^193^ Cells expressing NRP1 in the olfactory epithelium and endothelial cells of the olfactory bulb were also found to be infected by the virus.^155^ The host protease furin cleaves spike protein to produce a polybasic Arg-Arg-Ala-Arg C-terminal sequence on S1, which conforms to a C-end rule motif that binds to cell surface NRP1 and NRP2 receptors, but not the ACE2 receptor.^194^ Thus, NRP1 may serve as a host factor for SARS-CoV-2 infection and a candidate therapeutic target for the treatment of COVID-19. The binding occurs through the b1/b2 domain on the NRP1 receptor with the polybasic amino acid sequence 682RRAR685.^195^ A monoclonal antibody targeting NRP1 has been developed,^155^ but no challenge results have been reported in mouse models to date (Table 1).

#### hDPP4, AGTR2, ANPEP and ENPEP

MERS-CoV is another coronavirus that is highly pathogenic in humans, causing severe disease and sometimes lethal lower respiratory tract infection.^196,197^ Dipeptidyl peptidase 4 (DPP4), also known as CD26, has been identified as a functional receptor for MERS-CoV strain EMC.^198^ As an extracellular peptidase, hDPP4 was detected on the surface of different types of cells such as the liver, capillaries, lungs, kidneys, smooth muscle, and in the immune system.^199–201^ Nevertheless, the homolog of mouse DPP4 (mDPP4) does not play the same role.^202^ Mice transduced with adenoviral vector expressing hDPP4 could be infected,^203^ while hDPP4-transgenic or knock-in mice^204,205^ were susceptible to MERS-CoV infection and developed fatal disease. There is currently insufficient evidence that hDPP4 might support the SARS-CoV-2 infection in vivo. An experiment using HeLa cells showed that SARS-CoV-2 virions do not bind to the DPP4 receptor,^175^ but another report^206^ did observe such binding. Based on the similar expression pattern with human ACE2, Qi et al.^156^ as well as Venkatakrishnan et al.^206^ inferred that hDDP4 may be a cellular receptor of SARS-CoV-2.

Studies have shown that angiotensin II receptor type 2 (AGTR2), alanyl aminopeptidase (ANPEP) and glutamyl aminopeptidase (ENPEP) may also be receptors of SARS-CoV-2. AGTR2 is a G-protein coupled receptor with high and specific expression in the lungs, and showing a greater binding affinity towards SARS-CoV-2 S protein compared to ACE2. AGTR2 is capable of interacting with ACE2, and gene expression analysis revealed that when ACE2 is significantly downregulated, there is concomitant upregulation of AGTR2.^207^

Since ANPEP is a known cellular entry receptor for several coronaviruses, such as human CoV-229E, as well as canine and feline CoV,^156,208^ scientists considered it a candidate receptor for SARS-CoV-2. However, there is only evidence that ANPEP has a similar expression patter in the conjunctiva with ACE2.^209^

ENPEP plays an important role in regulating blood pressure and remodeling blood vessels. Qi et al.^156^ found that the expression pattern of ENPEP was similar to that of the ACE2. However, further studies are necessary to confirm the function of ENPEP as an entry receptor, or co-receptor for SARS-CoV-2 infection, since there is no in vivo evidence (Table 1).

### Proteases that mediate viral entry of SARS-CoV-2

Transmembrane serine protease 2 (TMPRSS2), the lysosomal proteases cathepsin B and L, the endogenous serine protease furin, and the serine endopeptidase trypsin plays critical roles in the viral entry of SARS-CoV-2.^144,181^ TMPRSS2 in particular has been widely studied.^210,211^ SARS-CoV-2 infection requires the maturation of the spike protein, before entry into target cells. Two separate mechanisms of cleavage may be involved, including ACE2 cleavage, which might promote viral uptake, and SARS-S cleavage, which activates the S protein for membrane fusion.^212^ The arginine and lysine residues among ACE2 amino acids 697 to 716 are essential for cleavage by TMPRSS2.^212^

#### TMPRSS2

Several studies have shown that TMPRSS2 can activate and cleave the S protein of SARS-CoV-1 for membrane fusion.^210,213^ Mechanistic insights into this process of ACE2 cleavage and activation were reported later.^212^ TMPRSS2 was found to be expressed in the human respiratory system, including the subsegmental bronchial branches and lungs.^214^ Furthermore, single‐nucleus RNA sequencing revealed that there is higher TMPRSS2 expression in type II pneumocytes, similar to ACE2 expression.^114^ In agreement with this, TMPRSS2 was found to be co-expressed with ACE2 in several sites, such as in the transient secretory cells of subsegmental bronchial branches and enterocytes in the small intestine,^214^ which may explain why these organs are vulnerable to SARS‐CoV‐2 infection. Camostat mesylate, which acts as a TMPRSS2 blocker, can partially inhibit SARS-CoV-2 entry in CaCo-2 cells, indicating the essential role of TMPRSS2 as an entry molecule.^171^ Smoking and exposure to air pollution may cause several comorbidities damaging the lungs, and are associated with more severe COVID-19 disease. Interestingly, smoking is associated with increased expression of TMPRSS2 and ACE2 in the human lungs.^215^ Notably, a TMPRSS2 deficient mouse model exhibited decreased susceptibility to SARS-CoV-2.^212^ Overall, the available literature indicates that TMPRSS2 plays crucial roles in the SARS-CoV-2 infection and cell entry.

#### Cathepsin B, cathepsin L, furin. and trypsin

As main lysosomal proteases, cathepsins B and L are commonly found in lysosomes and endosomes. They are responsible for monitoring the function of lysosomes^216,217^ and are commonly associated with aging neurons.^218^ Recently, it was reported that cathepsins B and L take part in SARS‐CoV‐2S protein priming, and are critical proteases for SARS‐CoV‐2 entry into HEK 293/hACE2 cells.^219^

A furin-like cleavage site was detected at the SARS‐CoV‐2 S1/S2 subunit,^220^ which is not present in other types of SARS coronaviruses. The ability of furin to cleave the S protein is thought to be the reason for the increased binding affinity of SARS‐CoV‐2 for the ACE2 receptor.^221^

As a serine endopeptidase, trypsin is highly expressed in respiratory and gastrointestinal cells. Recent reports indicate that trypsin may also be one of the proteases that aid SARS‐CoV‐2 entry.^181^ Bertram S et al.^211^ identified that human airway trypsin‐like protease could cleave and activate SARS‐CoV-1 S protein, which might cause viral spread in humans, but this finding requires further confirmation. Other common host factors, such as CSNK2B, GDI2, SLC35B2, DDX51, VPS26A, ARPP-19, C1QTNF7, ALG6, LIMA1, COG3, COG8, BCOR, LRRN2 and TLR9 may also regulate SARS-CoV-2 infection, and were discussed systematically in a recent review.^222^

## Animal models of COVID-19

### Non-human primate

Non-human primates share great similarity with humans in terms of physiological characteristics and immune regulation. Intensive efforts have been made to develop COVID-19 disease models in NHPs. Rhesus macaques (*Macaca mulatta*), Cynomolgus macaques (*Macaca fascicularis*), African green monkeys (*Chlorocebus sabaeus*), Baboon (*Papio hamadryas*) and common marmoset (*Callithrix jacchus*) have been employed as models of SARS-CoV-2 infection, whereby the first two are the most common selection (Table 2). It is worth mentioning that NHPs are facing enormous demand and soaring costs, so it may be necessary to find alternative models.

**Table 2:** SARS-CoV-2 non-human primate models

Animal species	Strains and inoculation	Outcomes				References
		Clinical signs	Pathogenesis	Virus loading	Virus shedding	
R. macaque (young to old)	WA1-2020, CN1, HB-01, USA-WA1/2020 Victoria/01, 3 × 10^3^ to^5^ × 10^6^ PFU, by ocular, oral, intranasal, intratracheal, aerosol, or combination of several above routes	Mild fever, weight loss, reduced appetite, and hypoxia, asthenia, decrease in platelet counts, transient neutropenia and lymphopenia	Pneumonia, pulmonary discoloration, consolidation, hyperemia, infiltrates, glass opacity, hemorrhage scar, and necrosis; liver and spleen lesions	Viral loads were detected in nasal, oral, throat, rectal swabs, BAL, pharynx, trachea, lung tissues, liver, spleen, paratracheal lymph nodes and blood	1–26 dpi	Munster et al.^224^;
						Gao et al.^55^
						Lu et al.^223^
						Blair et al.^228^
						Johnston et al.^226^
						Singh et al.^229^
C. macaque (Young to old)	USA-WA1/2020, Victoria/01/2020, BetaCoV/Munich/BavPat1/2020, 4.86 × 10^4^ to 5 × 10^6^ PFU, by aerosol, by I.N., I.T. route, or combination of several above routes	Weight loss, occasional reported fever and nasal discharge and elevated levels of liver-related enzymes	Diffuse alveolar damage (DAD), pulmonary discoloration, consolidation, infiltrates, endothoracic adhesion, glass opacity, liver and spleen lesions	Viral loads were detected in nasal, throat, OP, NP, rectal swabs, BAL, trachea, bronchus, lung tissues, spleen, ileum, feces, and blood, lower viral load in OP swabs compared to RMs and AGMs trachea	1–21 dpi	Rockx et al.^225^
						Lu et al.^223^
						Johnston et al.^226^
Africa green monkey (3 to 16 years)	USA-WA1/2020, INMIl-Isolate/2020/Italy, 2 × 10^3^ to 2.3 × 10^5^ PFU by aerosol, I.T. or I.N., or combination of the above routes	Severe respiratory distress, fever, decreased appetite, hypercapnia, Elevated liver-related enzymes, increased monocytes, transient lymphocytopenia and thrombocytopenia	Severe pulmonary consolidation and infiltration, extensive pulmonary lesions; Pulmonary discoloration, opacity, hyperemia and hemorrhage, pleural adhesions, and bronchointerstitial pneumonia	High quantities of viral RNA in respiratory tracts. detectable viral loads in BAL fluid	2–57 dpi (22 days post re-challenge)	Woolsey et al.^227^
						Blair et al.^228^
						Johnston et al.^226^
Common marmosets (adult to old)	USA-WA1/2020, or another isolated SARS-CoV-2 strains, 1 × 10^6^ PFU by I.N.	Mild fever	Slight Pulmonary infiltration	Low level of virus loads in nasal swab lung homogenate and blood	2–21 dpi	Lu et al.^223^
						Singh et al.^229^
Baboon (young to old)	USA-WA1/2020, 1.05 × 10^6^ PFU, by multi-routes of ocular, I.N., and I.T.	Progressive interstitial and alveolar pneumonitis	Pulmonary discoloration, infiltration, bronchiolization and syncytial cells	Detectable viral loads in buccopharyngeal, and rectal swab, BAL	1–17 dpi	Singh et al.^229^

*I.N*. Intranasal, *I.T*. Intratracheal, *BAL* bronchoalveolar lavage, *OP* oropharyngeal, *NP* nasopharyngeal

Rhesus macaques exhibit mild clinical disease and supporting high level of viral replication in the respiratory tract similar to humans. Mild fever, body weight loss, decreased appetite and hypoxia are common reported symptoms. Occasionally, asthenia, decrease in platelet counts, transient neutropenia and lymphopenia were also reported.^223^ Rhesus macaques display several histopathological lesions similar to those observed in patients, such as pulmonary discoloration, consolidation, hyperemia, glass opacity, infiltrates, hemorrhage, scar, necrosis and interstitial pneumonia.^224^ Lesions in the liver and spleen were reported as well. Although Rhesus macaques COVID-19 disease model recapitulates human symptoms most closely,^223^ some typical clinical symptoms, including acute respiratory distress syndrome, were not observed, which limits its application for mode detailed studies of COVID-19.

Cynomolgus macaques exhibit limited clinical symptoms, including mild fever and weight loss, nasal discharge and elevated levels of liver-related enzymes when challenged with SARS-CoV-2 via the intranasal or intratracheal route.^129,131–133,223^ Pulmonary consolidation is a common histopathological lesion in Cynomolgus macaques,^225^ as is observed in clinical patients. Another pathological change exhibited in COVID-19 patients, diffuse alveolar damage (DAD), was also observed in this animal model.^225^ In addition to the respiratory system, lesions in the liver and spleen also were observed in Cynomolgus macaques.^223^ Similar to Rhesus macaque, high levels of viral RNA were detected in the respiratory tract of challenged Cynomolgus macaques, even though they had lower viral loads and shorter duration of viral shedding.^226^

Several clinical signs were also seen in challenged African green monkeys, including transient fever, decreased appetite, hypercapnia, lymphocytopenia and thrombocytopenia, elevated liver-related enzymes, increased monocytes,^134,226–228^ and crucially, acute respiratory distress syndrome (ARDS).^228^ ARDS, the common and often fatal characteristic sign of severe COVID-19, is sustainably observed in aged African green monkey, while being difficult to replicate in other NHPs. African green monkeys, especially aged animals, are a quite useful model for mirroring severe disease manifestations, notably ARDS.^228^ Furthermore, viral pneumonia, severe pulmonary consolidation with hemorrhage and infiltration, extensive pulmonary lesions and gastrointestinal abnormalities were also observed in infected animals. Common histopathological lesions include pulmonary discoloration, opacity, bronchiolization, hyperemia and pleural adhesions.^227^ Compared to Rhesus macaques, African green monkeys exhibit more severe consolidation and edema of lung lobes.^226^

Compared with macaques, baboons exhibited more severe histopathological lesions, prolonged viral RNA shedding, substantially more lung inflammation,^229^ as well as showing age-related effects. By contrast, no or only very mild clinical symptoms (mild fever) were reported in common marmosets inoculated with SARS-Cov-2.^229^ Milder interstitial and alveolar pneumonitis were reported in marmosets, compared to in macaques or baboons.^229^ It is thought to be less susceptible to COVID-19 infection^223,229^ (Table 2). In sum, rhesus macaques are the most popular NHPs for COVID-19 disease because they are commercially available and manifest the clinical symptoms quite well. Cynomolgus macaques usually present pulmonary consolidation but show weak clinical symptoms. In contrast, African green monkeys generally exhibit severe symptoms, but their scarcity greatly limit the usage. Nevertheless, natural protective immunity, such as innate as well as humoral and cellular immune responses, can be induced in these NHPs.

### Mouse models

#### Stably inherited genetically modified mouse models

Mice and other rodents are the most widely used experimental animals. However, wild-type rodents are not spontaneously permissive for SARS-CoV-2 infection, because the virus can efficiently bind human ACE2 (hACE2), but not mouse *Ace2* (m*Ace2*). The expression of hACE2 was found to be related to cardiovascular and pulmonary diseases^230^ and it was also found to be a receptor of various coronaviruses including SARS-CoV-1^146^ and SARS-CoV-2.^181^ Stably inherited genetically engineered mouse models expressing hACE2, which results in great susceptibility, are critical for the preclinical evaluation of vaccines and drugs against SARS-CoV-2.

Several genetically modified hACE2 mouse models, driven by specific promoters and established via precision knock-in or random transgenic technologies (Table 3), have been adapted for research on cardiovascular disease and coronavirus infection.^114,138,231–233^ These mouse models have various backgrounds and can achieve stable expression of hACE2 in multiple organs. However, differences of hACE2 expression patterns in mouse models, different SARS-CoV-2 strains, as well as diverse infection routes and viral doses, lead to different clinical manifestations and pathological changes (Table 4). SARS-CoV-2 is transmitted through the respiratory tract, so most studies in Table 4 rely on the intranasal route to infect the model animals with SARS-CoV-2, except for hACE2-KI mice which were infected through intragastric inoculation.^114^ Unlike the upper respiratory tract signs (such as cough and dyspnea) that humans are most likely to develop after infection with SARS-CoV-2,^234^ weight loss is the most obvious and important clinical sign in mice. However, impaired lung function was observed in certain HFH4-hACE2 and K18-hACE2 mouse infection models (the background for these transgenic mice, see Table 3), including respiratory distress,^235^ markedly abnormal lung biomechanics^236^ and labored breathing.^163^ SARS-CoV-2 infection is usually confined to the respiratory tract in most hACE2 mouse models, but the brain can also be a target organ, suggesting nervous system invasion secondary to respiratory infections.^114,163,235,236^ Surprisingly, the SARS-COV-2 virus with broad organ tropism replicated in K18-hACE2 mice infected with intermediate viral titers (nCoV-WA1-2020).^163^ The animals exhibited systemic infection, with virus detectable in the nasal epithelium, trachea, lungs, heart, spleen, liver, kidneys, stomach, large intestine, small intestine and brain. In another K18-hACE2 mouse model, the infection of nasal epithelial cells, especially supporting sustentacular cells may be associated with anosmia as a major manifestation in mild disease.^50,164,237^ By intranasal inoculation of K18-hACE2 transgenic mice with higher viral doses (2 × 10^3^ and 2 × 10^4^ PFU) or lower doses (2 × 10^1^ and 2 × 10^2^ PFU), a model recapitulating both non-severe and severe COVID-19 infection were established, respectively.^238^

**Table 3:** The basic information of hACE2 inheritable genetic modified mice

Mouse models and background	Types	Promoter	hACE2 insertion sites	Copies of hACE2 gene	Expression of m*Ace2* or not	Expression profile of hACE2 genes	References
hACE2-KI, with C57BL/6 background	Humanized	Endogenous promoter of m*Ace2*	Chr X, GRC m38.p6	1 copy for heterozygote	No	Expressed in liver, spleen, lung, kidney, small intestine, brain and ovary, confirmed at mRNA level; expressed in lung, kidney and liver, confirmed by Western blotting; Expressed predominantly in CC10 + Clara cells, surfactant protein C positive (SPC+) alveolar type II cells, identified by immunofluorescence staining analysis	Sun et al.^114^
HFH4-hACE2, with C3B6 background	Transgenic	lung ciliated epithelial cell hepatocyte nuclear factor-3/ fork head homologue 4 promoter	Random insertion	Not specified	Yes	Mainly in lung, brain, liver, kidney, and gastrointestinal tract had varying levels of hACE2 expression, confirmed at mRNA level, no identification at protein and cell level	Menachery et al.^231^
K18-hACE2, with C57BL/6	Transgenic	human cytokeratin promoter, K18	Random insertion	4 to 10 copies tested by Q-PCR	Yes	Airway epithelial, lung, heart, brain, liver, kidney, spleen, duodileum and colon, confirmed at mRNA level, no identification at protein and cell level	McCray Jr et al.^232^
Tg hACE2 mouse, with ICR background	Transgenic	Mouse *Ace2* promoter	Random insertion	Not specified	Yes	Lung, heart, kidney and intestine, confirmed at mRNA level, no identification at protein and cell level	Yang et al.^233^

**Table 4:** SARS-CoV-2 models established with stably inherited genetically modified mice

Animal models	Inoculation	Outcomes				References
		Clinical signs	Pathogenesis	Tissue tropism	Virus shedding	
(1) hACE2-KI C57BL/6 mice	Beta-CoV/wuhan/AMMS01/2020; 4 × 10^5^ PFU, I.N. route	No obvious clinical symptoms, but 10% of weight loss in older mice (30 wks) on 3 dpi; less weight loss in young (4.5 wks) mice	Interstitial pneumonia in young and aged hACE2-KI mice, inflammatory cell infiltration, alveolar septal thickening, and distinctive vascular system injury; More lesions were observed in older hACE2-KI mice. CC10 + Clara cells are the major target cells of SARS-CoV-2 along the airway	Lung, trachea and brain were main target organs infected, but in feces of older mice, high titer of virus was detected. In neuron, astrocyte, and microglial cells	On 6 dpi, 10^7^-10^8.5^ copies/g virus titer were detected in young and older mice, older mice have higher virus loading	Shi et al.^24^
(2) hACE2-KI C57BL/6 mice	Beta-CoV/wuhan/AMMS01/2020; 4 × 10^6^ PFU, intragastric route	No obvious clinical symptoms in young mice	interstitial inflammation, with alveolar septal thickening	Lung and trachea	On 5 dpi, 3 × 106 copies/g virus titer	Shi et al.^24^
(3) HFH4-hACE2 mice	IVCAS6.7512; 3 × 10^4^ TCID_50_, I.N. route	One mouse (1/24) showed a rapid body weight decrease with dyspnea, 4/24 mice died, 4/24 mice showed noticeable body weight loss, respiratory distress, and neurological symptoms	Moderate interstitial pneumonia appeared from 3 dpi, some mice suffered from more severe pneumonia on 5 and 7 dpi, multifocal lesions, inflammatory cells at peri-bronchial and peri-vascular infiltration and fibroblast hyperplasia with exudation of fibrin and protein edema in some alveoli, even dissolved and necrosis	Lung, eyes, brain, heart	Virus detected on 1, 3, 5, 7 dpi, the highest virus loading in lung ranged from 9.4 × 10^3^ to 9.1 × 10^5^TCID_50_ per gram of tissue	Jiang et al.^235^
(4) Transgenic hACE2 ICR mice	SARS-CoV-2 strain HB-01, 10^5^TCID_50_, I.N. route	Up to 8% of weight loss on 5 dpi; gross lesions with focal-to multifocal dark-red discoloration in some of the lung lobes	Moderate interstitial pneumonia, thickened alveolar septa, infiltration of inflammatory cells, an accumulation of inflammatory cells in partial alveolar cavities; coalescing interstitial pneumonia with diffuse lesions	In lung, and possibly in intestine	Virus detected on 1, 3, 5, 7 dpi, the peak virus loads in lung reached 1 × 10^6.77^ copies per gram of tissue on 3 dpi	Bao et al.^165^
(5) K18-hACE2 mice	SARS-CoV-2 Hong Kong/VM20001061/2020; 8 × 10^4^ TCID_50_, I.N. route	exhibited variable clinical symptoms on 5 dpi, including eye closure, piloerection, respiration; loss around 10% body weight	Had alveolar proteinaceous debris, interstitial inflammatory cell infiltration, and alveolar septal thickening	Lung	Virus detected on 5 dpi, 1 × 10^5^ PFU	Moreau et al.^240^
(6) K18-hACE2 mice, strain C57BL/6J	2019n-CoV/USA_WA1/2019, 2.5 × 10^4^ TCID_50_, I.N. route	Had marked weight loss, lost about 25% on 7 dpi; plasma bicarbonate noticeably increased; markedly abnormal lung biomechanics on 7 dpi	progressive and widespread viral pneumonia with perivascular and pan-alveolar inflammation, immune cell infiltration, edema, and lung consolidation	Lung, heart, brain, kidney, spleen, duodileum, colon and serum	Virus detected on 2, 4, 7 dpi; the highest infectious virus in lung, 107 or so PFU on 2 dpi, and virus genome copies 10^8^ or so copies/g on 2–7 dpi	Winkler et al.^236^; Yinda et al.^163^

Lung histopathology check indicated that the hACE2 mouse model is capable of mimicking pneumonia associated with severe SARS-COV-2 infection, with diffuse alveolar damage,^237^ interstitial pneumonitis^114,163,235,239^ and inflammatory or lymphocytic infiltrates.^114,163,235,236,239–241^ Notably, the hACE2-KI mouse infection model exhibited distinct vascular system injury and pathological progression related to the age of the mice.^114^ Moreover, the thrombosis characteristic of critical illness was observed in the K18-hACE2 mouse infection model.^164^ At present, most studies on immunological processes that influence COVID-19 in mouse models are based on K18-hACE2 mice, which reflect changes in the immune response of the lungs. In particular, several chemokines (CCL2, CCL3, CCL4, CXCL1, and CXCL10) and inflammatory cytokines (TNF*α*, IL-6, and G-CSF) associated with the severity of COVID-19 disease in humans are highly expressed in these models.^221,236,242,243^ In aged mice, the interferon and adaptive antibody responses to the SARS-CoV-2 challenge are significantly impaired, resulting in more effective virus replications and severe disease manifestations.^244^ Importantly, these clinically relevant pathological findings are beneficial for studying the pathogenesis of SARS-CoV-2 in vivo.

#### Mouse models sensitized by Ad5-hACE2 or AAV-hACE2 transduction

Because the mouse ACE2 receptor does not support viral binding, wild-type mice are not permissive to SARS-CoV-2 infection.^170,245,246^ To sensitize the wild-type mice, adeno associated vector or adenovirus 5 expressing hACE2 (AAV-hACE2, Ad5-hACE2) were used to transduce mice intratracheally, causing hACE2 expression in lung tissues, which enabled viral entry and infection (Table 5). Different from stably inherited transgenic mice, these sensitized mice express hACE2 in respiratory tract, especially in the lungs, and the expression pattern is different from both transgenic mice and human.^41,247–250^ Upon infection with SARS-CoV-2, the virus replicates in the lungs of hACE2 mice for several days to two weeks, depending on the mouse strain used (Table 5).^251^ Immunodeficient mice such as IFNAR^−/−^ and STAT1^−/−^ presented delayed virus clearance, while in immunocompetent mice quickly cleared the virus.^252^ The challenged C57BL/6 mice transduced with AAV-hACE2 developed the characteristics of moderate interstitial pneumonia, including inflammatory infiltration in peri-bronchia, diffuse infection within alveolar epithelia, expansion of pulmonary infiltrating myeloid-derived inflammatory cells as well as the recruitment of monocyte-derived macrophages and inflammatory cells.^252^ Viral infection in the BALB/c or C57BL/6 mice transduced with Ad5-hACE2 induces severe inflammation in the upper and lower respiratory tract, including the infiltration of inflammatory cell from perivascular to interstitial zones necrosis, the aggravation of alveolar edema, vascular congestion and hemorrhage.^179^ Accompanied by the pathological changes, there were significant clinical signs in the mice transduced with Ad5-hACE2, while there were no significant clinical signs in the mice transduced with AAV-hACE2, which had a lower viral load.^252^

**Table 5:** Mouse models sensitized by Ad5-hACE2 or AAV-hACE2 transduction

Animal models	Strains and inoculation	Outcomes				References
		Clinical signs	Pathogenesis	Infected organs	Virus shedding	
(1) AAV-hACE2 wild-type C57BL/6 mice	Using nCoV-WA1-2020 strain, with the dosage of 1 × 10^6^ TCID_50_ and I.N. challenge route	No significant weight changes or death	Mild diffuse peribronchial infiltrates, diffuse infection within alveolar epithelia; expansion of pulmonary infiltrating myeloid-derived inflammatory cells; inflammatory monocyte-derived macrophages and inflammatory cells	Just in lung, and virus cleared on 7 dpi	Virus shedding were checked on 2, 4, 7, and 14 d.p.i., and increased 200 folds of virus RNA copies, *V.S*. mock group; increased 10^4^ PFU VS. mock group	Israelow et al.^252^
(2) AAV-hACE2 IFNAR^−/−^ C57BL/6 mice	Using nCoV-WA1-2020 strain, with the dosage of 1 × 10^6^ TCID_50_ and I.N. challenge route	No significant clinical sign	Loss of recruitment of Ly6C^hi^ monocytes and monocyte-derived macrophages; complete loss of activation of CD4^+^, CD8^+^, or NK cells; robust recruitment of neutrophils	Just in lung, virus still be detected on 15 dpi	Virus shedding on 2, 4, 7, and 14 dpi, and increased 2000 folds of virus RNA copies, *V.S*. mock group; increased 10^6^ PFU *V.S*. mock group	Israelow et al.^252^
(3) AAV-hACE2 IRF3/7^−/−^ C57BL/6 mice	Using nCoV-WA1-2020 strain, with the dosage of 1 × 10^6^ TCID_50_ and I.N. challenge route	No significant clinical sign	Loss of recruitment of Ly6C^hi^ monocytes and monocyte-derived macrophages; reduced activation of CD4+, CD8+, or NK cells	Just in lung, virus still be detected on 15 dpi	Virus shedding increased 200 folds of virus RNA copies, *V.S*. mock group; increased 10^5^ PFU *V.S*. mock group	Israelow et al.^252^
(4) Ad5-hACE2 transduced BALB/c mice	Using an isolated strain, with the dosage of 1 × 10^5^PFU and I.N. challenge routes	Weight loss, ruffled fur, hunching, and difficulty breathing	Perivascular to interstitial inflammatory cell infiltrates, necrotic cell debris; alveolar edema, increased vascular congestion and hemorrhage	High titers in lung tissue and gradually declined	Virus loads were detected in 1–10 dpi	Sun et al.^179^
(5) Ad5-hACE2 transduced C57BL/6 mice	Using an isolated strain, with the dosage of 1 × 10^5^PFU and I.N. challenge routes	Weight loss	similar to those examined in Ad5-hACE2 transduced BALB/c mice	Highest virus titers in lung at 1–2 dpi and gradually declined	Virus loads were detected in 1–10 dpi	Sun et al.^179^
(6) Ad5-hACE2 transduced IFNAR^−/−^ C57BL/6 mice	Using an isolated strain, with the dosage of 1 × 10^5^PFU and I.N. challenge routes	Weight loss	None	Delayed virus clearance in lung	Virus loads were detected in 2, 4 and 6 dpi	Sun et al.^179^
(7) Ad5-hACE2 transduced IFNγ^−/−^ C57BL/6 mice	Using an isolated strain, with the dosage of 1 × 10^5^PFU and I.N. challenge routes	Weight loss	None	Delayed virus clearance in lung	Virus loads were detected in 2, 4 and 6 dpi	Sun et al.^179^
(8) Ad5-hACE2 transduced STAT1^−/−^ C57BL/6 mice	Using an isolated strain, with the dosage of 1 × 10^5^PFU and I.N. challenge routes	Greater weight loss	Enhanced inflammatory cell infiltration into the lungs	Delayed virus clearance in lung	Virus loads were detected in 2, 4 and 6 dpi	Sun et al.^179^

The immune response is crucial for antiviral defense and lymphopenia is widespread in severe cases of SARS-CoV-2.^253^ Thus, the models based on immunocompromised mice can mimic the severe outcomes and features of SARS-CoV-2 infection in humans. In the IFNAR knockout mice transduced with AAV-hACE2, the recruitment of Ly6Chi monocytes and monocyte-derived macrophages was abolished, the activation of CD4^+^, CD8^+^ or NK cells was inhibited, and the accumulation of neutrophils was increased instead.^254^ The inflammatory characteristics of IRF3/7^−/−^ mice are similar to IFNAR^−/−^ mice transduced with AAV-hACE2, but the viral load is significantly higher in the latter (Table 5). Ad5-hACE2 transduced STAT1^−/−^ mice are the most permissive to SARS-CoV-2.^179^ Overall, the outstanding advantage of AAV- or Ad5- transduced mice model is the rapid acquisition of susceptible animal models for emergency needs, but such disease models can only mimic limited physiological features of SARS-CoV-2 infection.

#### Models based on mouse-adapted strains of SARS-CoV-2

It is of great significance to study the pathogenicity of viruses by analyzing the emerging mutations of mouse-adapted strain.^255,256^ Huang et al. passaged the SARS-CoV-2 WuHan-Hu-1 strain in 1-year-old BALB/c mice. Lung homogenates were used to intranasally inoculate young mice, and after 11 rounds, the mouse-adapted virus strain WBP-1 was generated. Compared to its ancestral strain, WBP-1 showed increased infectivity in BALB/c mice and caused more severe interstitial pneumonia. Sequence analysis revealed that the Q498H mutation emerged after only one passage, and the Q493K mutation occurred in passage 5, which likely contributed to the high pathogenicity of WBP-1 in mice.^257^ Further, the two mutations are both located in the RBD, and significantly increased its binding affinity for mouse ACE2. The study tentatively found that the TLR7/8 agonist resiquimod was able to protect mice against WBP-1 challenge, indicating that this mouse-adapted model may be used as a tool for investigating COVID-19 therapies.

The proposed model should s recapitulate the main features of SARS-CoV-2 infection in humans, supporting efficient viral replication in both the upper and lower respiratory tracts.^258^ Wang et al. intranasally inoculated used 4- to 6-week-old female BALB/c mice with SARS-CoV-2 HRB26, and after for 14 passages the mouse-adapted strain HRB26M was generated. Mild pathological changes were observed in wild-type BALB/c mice when challenged with this strain, demonstrating that SARS-CoV-2 successfully adapted to infect the upper and lower respiratory tract of young BALB/c mice.^259^ Gu et al. reported a more rapid method, which they used to generate the mouse-adapted strain named MACSP6, after only six passages in BALB/c mice.^260^ The key RBD mutation (N501Y) was observed in this strain. After an additional 30 passages, a more virulent mouse-adapted strain named MACSP36 was generated, which elicited typical respiratory symptoms in 9-month-old mice, especially tachypnea was exhibited in all moribund animals.^256^

In addition to accumulating mutations through serial passages, precise reverse genetic technology was employed to rapidly establish mouse-adapted strains (Table 6). Dinnon et al.^261^ used reverse genetics to remodel the S and *mAce2* binding interface, resulting in a recombinant virus, named SARS-CoV-2 MA. This virus utilized *mAce2* for entry, and showed more clinically relevant phenotypes than those seen in hACE2 transgenic mice, demonstrating its utility. With further passaging, the strain resulted in a linear decrease of body weight in challenged BALB/c mice. Furthermore, 10-week-old BALB/c mice challenged with the mouse-adapted strain MA10 exhibited a loss of pulmonary function, accompanied by increased morbidity and mortality. A study also reported that 10-week-old C57BL/6 J mice exhibited less severe disease when infected^262^ (Table 6).

**Table 6:** Models based on mouse-adapted strain of SARS-CoV-2

Animal models	Strains & Inoculation	Adaptive mutations	Outcomes				References
			Clinical signs	Pathogenesis	Infected organs	Virus shedding	
(1) Wild-type BALB/c, nine-month-old	Original strain was IME-BJ05 passaged for 6 times in old BALB/c mice; Mouse adaption strain was named MASCp6; Inoculation dosage was 1.6 × 10^4^ PFU with I.N. routes	5 nucleotide mutations distributed within ORF1ab, S and N gene; the key mutation in A23063T resulting in N501Y substitution in RBD of S protein	No significant weight changes or death	Mild to moderate pneumonia, interstitial pneumonia, vessel injured, with adherent inflammatory cells; self-recovered on 5 dpi	The most wildly infected organs: Lung, trachea, feces, intestine, heart, liver, spleen and brain on 3 dpi	Virus shedding was detected on 3, 5, 7 dpi; maximal virus titer 10^10^ copies/g in lung on 3 dpi	Gu et al.^260^
(2) Wild-type BALB/c, six-week-old	Original strain was IME-BJ05 passaged for 6 times in old BALB/c mice; Mouse adaption strain was named MASCp6; Inoculation dosage was 1.6 × 10^4^ PFU with I.N. routes	5 nucleotide mutations distributed within ORF1ab, S and N gene; the key mutation in A23063T resulting in N501Y substitution in RBD of S protein	No significant weight changes or death	mild to moderate pneumonia, but much milder than the old mice	The most wildly infected organs were lung, trachea, feces, intestine, heart, liver and kidney observed on 5 dpi	Virus shedding was detected on 3, 5, 7 dpi; maximal virus titer 10^11^ copies/g in lung on 3 dpi, almost 10 folds higher than older BALB/c mice	Gu et al.^260^
(3) Wild-type BALB/c, 12-week-old	Original strain was SARS-CoV-2; Mouse adaption strain was named SARS-CoV-2 MA; Inoculation dosage was 1.0 × 10^5^ PFU with I.N. routes	By revers genetics technology to make the mouse adaption strain, in which including RBD Q498T and P499Y substitutions	No weight loss or other clinical signs; loss of pulmonary function	No pathologic changes were described	Mainly infected lung, small nasal virus in nasal turbinate	Virus detected on 2, 4 dpi; maximal PFU appeared on 2 dpi, but almost cleared on 4 dpi	Dinnon et al.^261^
(4) Wild-type BALB/c, one-year-old	Original strain was SARS-CoV-2; Mouse adaption strain was named SARS-CoV-2 MA; Inoculation dosage was 1.0 × 10^5^ PFU with I.N. routes	By revers genetics technology to make the mouse adaption strain, in which including RBD Q498T and P499Y substitutions	Weight loss near to 10% on 3 dpi, then recovered; presenting more severe loss of pulmonary function than young BALB/c mice	No. pathologic changes was described	Mainly infected lung, small nasal virus in nasal turbinate	Virus detected on 2, 4 dpi; The maximal PFU over 10^6^ appeared on 2 dpi, but many mice almost cleared on 4 dpi	Dinnon et al.^261^

#### Humanized mouse models with engrafted human tissues or cells

The human immunological mechanisms defining the clinical outcome of SARS-CoV-2 infection remain elusive. Although the animal models susceptible to SARS-CoV-2 have been available,^131,133,210,220,230,240^ an in-depth understanding of the immunopathogenesis has been hindered by the vast difference in both immune responses and lung environments between humans and animal models.^263,264^ That said, the SCID-hu lung mouse model has been proved to be an alternative approach to resolve this problem as it shows rapid virus replication, severe lung damage, and robust pro-inflammatory responses.^265^ To establish this model, human fetal lung tissues were surgically grafted into the dorsal skin of SCID mice and were allowed to grow for about 8 weeks followed by SARS-CoV-2 virus challenge by direct injection into the engrafted lung tissues.^265^

However, optimal humanized mouse models should carry not only human functional lung tissues but also the human immune system, thereby more precisely recapitulating human immunopathology. A mouse model co-engrafted with human fetal lung tissues and a myeloid-enhanced human immune system was created recently,^263^ which could serve as a better model than the one solely engrafted with human fetal lung tissues to identify cellular and molecular correlates of lung protection against SARS-CoV-2 infection. These mice exhibit severe inflammatory and histopathological phenotypes, which are associated with macrophage infiltration and differentiation and the upregulation of genes involved in the type I interferon signaling pathway.^263^ Taken together, engrafted mice models are an alternative and complementary approach to the currently used human lung organoid model^265^ for the studies on the immunopathology of SARS-CoV-2 infection. However, the limited supply of human tissues and immune cells makes the scale-up difficult. Moreover, the challenge route through direct injection is quite different from the clinical infection.

### Syrian hamster models

Syrian hamsters have been shown susceptibility to SARS-CoV-1 more than one decade ago, and were proven to be susceptible to variable SARS-CoV-2 isolates recently. In accordance with these findings there is great sequence similarity between human ACE2 and that of hamsters.^29,165,233,266–270^ Respiratory and pulmonary disease, accompanied by weight loss, occurs in hamsters upon infection.^271,272^ Weight loss is the main clinical sign which serves as the indicator of infection severity, but is reversed later. Viral load could be detected in the respiratory tract, including bronchial epithelia cells, type I and type II alveolar epithelial cells and macrophages, which are also targets in human lung tissues by SARS-CoV-2.^273–277^ Virus replication in these tissues reaches the peak at the early stage after infection, followed by a rapid decline. However, almost no viral RNA can be found in the spleen, kidneys, blood, duodenum and brain of infected hamsters, except in some extreme cases.^273^ Virus propagation is accompanied by inflammation, tissue damage,^278,279^ and lung abnormalities were found in SARS-CoV-2 challenged hamsters, including interstitial pneumonia, inflammatory cell infiltration, alveolar septal thickening and distinctive vascular system injury.^280^ Afterwards, a macrophage-dominated pulmonary immune response was induced.^278^ Sia et al. also observed the infiltration of CD3^+^ T lymphocytes and monocytes in the peribronchial region after infection.^279^ After lung damage and histopathological changes caused by virus replication, the viral load decreased rapidly and lung damage resolved in the next days of infection. The histopathological changes in arterial and venous endothelium of hamsters were consistent with endotheliosis in human SARS-CoV-2 infection.^273,281^

Some studies showed long term damage to other physiological systems including the olfactory sensory system, reproductive system and cardiovascular system.^282–287^ Cardiovascular pathologies were found in female hamsters with late-stage SARS-CoV-2 infection, including myocardial interstitial fibrosis, as well as thickening of ventricular walls and the interventricular septum.^284^ In addition, SARS-CoV-2 infection changes the serum lipid and metabolite profiles of the hamsters, which was also observed clinically in human patients.^284^ Another study revealed that SARS-CoV-2 infection caused reproductive problems in male hamsters, including acute decrease of the sperm count and serum testosterone, as well as reduced testicular size and serum sex hormone levels for months after infection.^288^ The circulating strain Omicron was found to cause similar changes, but vaccination protected male hamsters from the testicular damage.^288^

According to the clinical manifestations, SARS-CoV-2 causes more severe outcomes in males than in females, and sex differences of acute and chronic damage after infection were also studied in the hamster model.^289^ In addition, Osterrieder et al. revealed that the progression of SARS-CoV-2 in Syrian hamsters is age-dependent, since viral replication in the upper and lower respiratory tract was different according to the age of hamsters. Consequently, older hamsters exhibited more severe clinical signs, such as weight loss.^273^

The close contact transmission model was also tested in hamsters, demonstrating that cohousing contact with SARS-CoV-2-carrying hamsters is an efficient way to spread the disease. At the same time, it was found that mRNA-HB27-LNP has a prophylactic effect against SARS-CoV-2 transmission through close contact.^280^ It was also demonstrated that immunosuppressed hamsters are susceptible to low-dose virus inoculation, while developing more severe and prolonged disease.^290–292^ Brocato et al.^203,204^ exposed cyclophosphamide immunosuppressed and *Rag2* knockout hamsters to SARS-CoV-2 by the respiratory route to test this hypothesis.^293,294^ Although the ACE2 of hamsters can be used as a cell entry receptor by human SARS-CoV-2, some of the contact residues in hACE2 are not conserved, resulting in reduced susceptibility to infection.^295^ Halfmann et al.^296^ used the hACE2 transgenic hamsters to test the infectivity of the Omicron variant. The relevant studies using Syrian hamsters as animal models are summarized in Table 7.

**Table 7:** SARS-CoV-2 Syrian hamster models

Animal models	Strains and inoculation	Outcomes					References
		Clinical sign	Immunohistochemistry	Histopathology	Infected organs	Virus shedding	
(1) 4,5-week-old male hamsters	SARS-CoV-2 (Beta CoV/Hong Kong strain, with the dosage of 8 × 10^4^ TCID_50_ and I.N. challenge route	Weight loss	Viral antigen colocalized with mononuclear infiltration in lung, N protein was detected in bronchial epithelial cells at 2 dpi; Viral antigen was detected in the nasal epithelial cells and olfactory sensory neurons at the nasal mucosa	Inflammatory cells and consolidation in 15–35% lungs at 5 dpi, mononuclear cell infiltration with viral antigen at 2 and 5 dpi; 30–60% consolidation in the lungs at 7 dpi In nasal turbinate, moderate inflammatory cell infiltration; reduction in the number of olfactory neurons at nasal mucosal at 2 dpi and repaired at 14 dpi	In lung, kidney, nasal turbinate and duodenum with clearance on 7 dpi	Virus checked on 2, 5, 7 dpi, viral peak in lungs occurred at 2 dpi, no virus was detected on 7 dpi	Sia et al.^279^; Deng et al.^280^
(2) 4-weeks-old and 28 to 32- weeks old hamsters	UT-NCGM02 strain, the dosage of 1 × 10^5.6^ or 10^3^ PFU by intranasal and ocular routes	4-weeks-old hamster, the maximal weight loss of high dose infection occurred at 6 dpi; 28 to 32- weeks old hamsters, severe weight loss at 7 dpi of high dose infection and continued to lose weight for up to 14 dpi	Viral antigens were detected in bronchi, lungs, and nasal mucosa and olfactory epithelium on 3 and 6 dpi; No viral antigens in the brain were detected, no viral antigens can be detected at 10 dpi	Micro-CT analysis revealed lung abnormalities since 2 dpi including ill-defined, patchy ground glass opacity (GGO) in younger hamster; then developed into severe lung abnormalities in all infected animals; severe lung lesions occurred at 3 dpi	Virus detected in the respiratory organs of both higher or lower dose group in nasal turbinate, trachea and lungs at 3 dpi, no obvious difference between the inoculation doses at 3 dpi	Virus checked on 3,6,10 dpi, no virus was detected on 10 dpi	Imai et al.^277^
(3)8-10 weeks old male Syrian hamsters.	SARS-CoV-2 strain HK-13, Delta, and Omicron variants, 1 × 10^5^ PFU, I.N. route or direct intratesticular injection	Reduction in sperm count; decrease in serum testosterone and inhibin B levels; reduction of size and weight of testes	Vimentin and Deleted in Azoospermia Like (DAZL) protein, Sertoli cells expressed vimentin deformed and detached, cytoplasmic vacuolation degeneration, loss of cytoplasmic arms; germinal cells disarranged, detached or in form of multinucleated giant spermatocyte; spermatogonia; damaged seminiferous tubules	Expanded testicular interstitial space, edema, germ cell sloughing and severe testicular hemorrhage; interstitial mononuclear cell infiltration; severe seminiferous tubular necrosis, occasional neutrophils, and disordered germ cells arrangement, reduced layers of spermatogenic cell spectrum	Testes and lung	low viral loads were found in only a few testicular samples at 4, 7, 42 and 120 dpi	Li et al.^288^
(4) hACE2 transgenic hamsters	Using three isolates, B.1.617.2, B.1.1.529 and WA1/2020 D614G, with the dosage of 1 × 10^3^ PFU and I.N. challenge route	D614G isolate, marked weight loss within the 1st week; uniform mortality by 10 dpi; B.1.1.529, less weight loss and death	None	In hACE2 transgenic hamsters, lung infection, clinical disease and pathology with B.1.1.529 were milder than with historical isolates or other SARS-CoV-2 VOC	Nasal turbinate, lung	B.1.1.529, 1000- to 10,000-fold lower levels of infectious virus in the lungs at 3 and 5 dpi	Halfmann et al.^296^

### Ferret and mink models

Both ferrets and mink are members of the Mustelidae and are naturally highly susceptible to several human respiratory viruses, including SARS-CoV-1.^297,298^ Thus, it is reasonable to assume that SARS-CoV-2 infection could also be studied using these models.^299^ The susceptibility of minks was confirmed by a report on the numerous infections with SARS-CoV-2 across 40 farms in 2020.^300^ Upon infection, ferrets showed common clinical symptoms such as fever and mild respiratory symptoms, but no death was record.^299^ Virus replication was detectable in nasal washes, saliva, urine, and feces until the 8^th^ day post infection (dpi), usually peaking on the 3rd dpi and in some studies, declined gradually and completely disappeared by the 14th dpi.^243^ Ryan et al.^301^ infected ferrets with three SARS-CoV-2 dosages. In the 5 × 10^2^ PFU dosage group, one of six ferrets displayed viral RNA in the upper respiratory tract, while in the 5 × 10^4^ PFU dosage group, viral RNA could be detected in the upper respiratory tract of all ferrets until the 14th dpi. Intermittent positivity occurred from 14 to 21 dpi in the 5 × 10^6^ PFU dosage group.

Pathological examinations revealed mild bronchial interstitial pneumonia at 7 dpi with 5 × 10^4^ PFU of SARS-CoV-2,^301^ and no other clinical symptoms or deaths were observed. The lungs of infected ferrets may demonstrate mildly expanded alveolar septa and diffuse interstitial histiocytic pneumonia after infection with 5.4 × 10^5^ TCID_50_/ml SARS-CoV-2.^302^ In contrast, infected minks displayed moderate respiratory signs such as labored breathing, bronchiolitis, and diffuse alveolar damage (DAD),^303^ whereas some minks died of infection.

An outstanding usage of ferrets and mink is for transmission studies.^299^ Infection was reported following direct inoculation, direct contact and indirect contact, as well as transmission between infected and naïve ferrets and minks.^299^ Different from other animal models, SARS-CoV-2 can be detected in the nasal cavity of ferrets and they can be infected through indirect contact, indicating that ferrets and minks are capable of spreading virus, which simulates the transmission route of SARS-CoV-2 in humans.^24,304,305^

### Poultry and domestic animals

There are truly vast numbers of poultry and domestic animals in the world. Moreover, they are essential to human life, and have daily close contact with humans. It is therefore necessary to address their potential susceptibility to SARS-CoV-2.^24^ Cats, dogs, chickens, ducks, pigs^24,306^ and sheep^305^ have been tested as potential animal models of COVID-19.^41^ Sub-adult outbred domestic cats aged 6–9 months were intranasally inoculated with 10^5^ PFU of SARS-CoV-2 strain CTan-H and viral RNA was detected in the upper and lower respiratory tract, but not in the lung samples.^24^ However, no clinical signs were reported. In addition, cats are susceptible to airborne transmission. It should be noted that cats are not a standard animal model, with limited immunological resources and possible injury to laboratory technicians.^24^ Due to their aggressiveness, cats are especially difficult to handle in biosafety level-3 containment.^41^

Available studies show that dogs (*Canis lupus familiaris*) exhibit only very mild susceptibility to SARS-CoV-2 infection, and no clinical signs were recorded, indicating that dogs are not suitable for the development of in vivo model.^24^ In addition, chickens, pigs and ducks were challenged with 10^5^ PFU of the CTan-H SARS-CoV-2 strain, but the lack of clear results or clinical signs indicates that they are not permissive to infection.^24^ The susceptibility of embryonated chicken eggs was also tested, but they could not support virus replication.^304^

As commonly farmed domestic ruminant animals, sheep were also recently investigated as hosts for SARS-CoV-2 infection,^305^ further expanding the range of animal models of COVID-19. However, experimentally challenged sheep only supporting limited virus replication according to nasal and oral swabs on 1 and 3 dpi, with virus detectable in the respiratory tract and lymphoid tissues on 4 and 8 dpi. Infection of naïve sentinel sheep may occur via respiratory droplets or aerosol. Interestingly, two SARS-CoV-2 isolates, B.1.1.7-like alpha VOC and its ancestral lineage A, were used to co-infect sheep, whereby the former outcompeted its ancestral lineage A,^305^ presenting experimental evidence in animal models that VOC alpha has stronger infectivity.

## Gaps of available models and future perspective

Animal models for SARS-CoV-2 infection have played crucial roles in the studies of infection mechanism, transmission, and immunity actions, which have fostered the development of COVID-19 vaccines and therapeutics during the pandemic.^41,307^ Each animal model has its own advantages and limitations. The available genetically modified mouse models were mostly established based on a single receptor gene, which has resulted in a failure to meet all the practical requirements. On the other hand, the global response to COVID-19 is now facing a new phase characterized by the emergence of several SARS-CoV-2 VOC and demands for studies on comorbidities^42^ as well as vaccine evaluation. It is there for necessary to discuss the strategy of developing the next generation of COVID-19 animal models for the future.

### Gaps of available animal models

1. The development of models that develop more severe disease may be needed. A common limitation of all the available animal models their limited ability to mimic the clinical features of COVID-19.^42^ The hamster model has emerged as one that more closely mimics moderate disease of humans,^279,284,287,308^ developing respiratory disease and displaying some other important clinical hallmarks found in patients after SARS-CoV-2 infection. However, it seems imperative to develop more tools to study the immunology if hamsters, since they expected to be an important animal model for severe disease. Such an animal model should present significant clinical symptoms, with the weight loss reaching at least 25%.^42^
2. Susceptible animal models supporting long duration of virus replication are still not widely available. The current SARS-CoV-2 models, including NHPs (Table 2), rodents (Tables 3, 4), and other models (Tables 5–7), only support viral infection for a short time, usually less than one week. This duration is not suitable for suboptimal doses of either a monoclonal antibody or a small molecule antiviral, and drug-resistant variants may emerge.^309^
3. It is necessary to develop models that recapitulate the effects of comorbidities on SARS-CoV-2 infection. patients with preexisting comorbidities show more severe symptoms when infected,^310,311^ but there is very limited knowledge on the effects of comorbidities on SARS-CoV-2 infection in animal models. Rodents are a better choice for such models than other species, since it is easier to establish models of SARS-CoV-2 in rats and mice with cardiovascular disease,^312^ cancer,^313^ and diabetes.^314^
4. It is well known that aged people that have a higher case fatality rate,^315^ incidence and more severe clinical characteristics. It is therefore necessary to develop animal models that mimic the effect of aging on SARS-CoV-2 infection and immunity.
5. Animal models that recapitulate the COVID-19 disease progression and the transition from mild to severe illness are urgently needed. With such models, it may be possible to identify novel biomarkers that better predict the course of human disease.^316,317^
6. Animal models for SARS-CoV-2 VOC will be essential,^318–320^ to evaluate pathogenicity and transmissibility, to assess whether the available vaccines and therapeutics can protect from VOC infection,^321,322^ and to further assess the potential risk of vaccine-associated enhanced respiratory disease (VAERD),^323^ especially in the context of mass vaccination and heterologous vaccination regimens.^324–326^
7. It may be necessary to develop animal models with Th1 and Th2 responses may be needed. Accordingly, models based on C57BL and BALB/c are both needed.^327,328^ A humanized ACE2 mouse model with BALB/c background has become available in China (https://www.gempharmatech.com).
8. Animal models with double and triple humanized gene combinations, rather than a single transgene^327^ are urgently needed for the next phase of studies. The obvious deficiencies of current mouse models include only using a single transgene, random integration with undetermined transgene copy numbers^233^ and controversial promoter selections, causing unnatural gene expression profiles and features not found in clinical patients.^235^ In addition to hACE2, more alternative receptors, co-receptors^144^ and primary antibody clearance receptors and host factors,^222^ such as TMRPSS2 and FcγRT were discovered. These prerequisites provide the possibility to establish advanced multigene humanized mouse models.

### Developmental strategies for the next generation of COVID-19 animal models

#### Genetically modified mice with single genes or multi-gene combinations

Fontela et al.^42^ pointed out some gaps of current animal models, finding that single hACE2 transgenic mice poorly mimic the clinical features of COVID-19.^327^ It is therefore necessary to develop mouse models with novel single genes or multi-gene combinations. Several new candidate receptors, as well as host proteases (Table 1), and host factors,^222^ have been found to play crucial roles in SARS-CoVp2 infection. Because mice are easier to genetically modify and are more widely used than other animal species, the modification of novel single or multiple gene(s) in mice should be the first choice. Novel potential receptors including CD147, ASGR1, KREMEN1 and Nrp1 may be worth trying in the construction of mouse models. An NSG immunodeficient mouse expressing the CD147 gene has been reported.^329^

Jarnagin et al.^327^ proposed a variety of multi-gene combination modes and implemented them step by step, including hACE2 and hTMPRSS2 double gene combination, hACE2 and hFCγRT double gene combination, or hACE2, hTMPRSS2 and hFCγRT triple gene combination. The authors also proposed the combination of hTMPRSS2 and hFCγRT.^327^ We assume that further humanizing CD147, ASGR1 and host protease hTMPRSS2, based on the available humanized hACE2-KI mice,^179^ may provide mouse models that can duplicate severe COVID-19 disease, which are urgently needed for future studies. On the other hand, antibody-dependent enhancement (ADE) poses a great threat to vaccine and antibody therapy. Yet, the methods to evaluate ADE in animal models are still needed to be explored. Recently, Okuya et al.^330^ reported that ADE could be mediated by two host factors, the Fcγ receptor and the complement component, C1q. Therefore, an FCγR and hACE2 double humanized mouse model would be a useful tool for ADE evaluation in vivo. However, it remains challenging to co-express multiple human genes in one mouse model.

Mice with T, B or NK cells deficiency are susceptible to a variety of viruses and support long duration of viral replication.^331–333^ Since one of the obvious defects of the current COVID-19 animal models is typically a limited duration of viral replication, they are not suitable for exploring whether drug-resistant variants may emerge. Therefore, either animal strains with natural immunodeficiencies or the experimental induction of immunodeficiencies by knockout of *Rag2* gene would be worth exploring, which may prolong the in vivo replication of SARS-CoV-2.^293,329,334^ Animal models supporting the long duration of SARS-CoV-2 infection may be used in the studies on the sequela of COVID-19, namely long COVID-19.^335^

An important finding is that older COVID-19 patients present more severe disease,^308,336^ as well as those with preexisting comorbidities^309^ such as cardiac disease,^337,338^ diabetes,^339–341^ and cancer. However, there is very limited knowledge on the effect of comorbidities in animal models. Available humanized or transgenic hACE2 mice can be used to construct mouse models suitable for the study of different complications by additional disease model induction or cross-breeding with available genetically models such as diabetic mice,^314^ aging mice,^342^ cardiovascular disease mice^343–345^ and tumor mice.

#### Selection of transgenic or knock-in strategy

Animal models humanized for the ACE2 receptor or other genes were generated by inserting hACE2 receptor or other foreign genes, driven by intrinsic the mouse promoter, into the locus of *mAce2* or analogous genes. The first advantage of such models over random insertion is that the expression pattern of the transgene closely mimics natural expression.^179,346–348^ Secondly, the interference of mouse orthologs is excluded, and thirdly, transgenes are integrated as single copy. However, intrinsic mouse promoters usually lead to a lower expression of transgenes, resulting in poor susceptibility and weak clinical symptoms (Table 4). The K18 promoter^164,235,242^ and HFH-4 promoter^349^ may result in high expression of transgenes, but the expression pattern is non-physiological.^235^ New models are increasingly being established using precision knock-in, rather than random insertion.^350,351^ Safe harbor sites, such as Rosa26^352,353^ and Hipp11^178,354^ are increasingly being preferred for the insertion of heterologous genes.^355^

Powerful promoters, such as the CAG promoter,^356–358^ may be employed instead of intrinsic mouse promoters, whereby the transgene expression cassette is still inserted into the locus of the respective mouse ortholog to remove interference while still guaranteeing strong expression. This strategy may be used to construct animal models of infection with other pathogens.

#### Genetic background of animal models

The genetic background has a strong effect on numerous characteristics of animal models,^359^ including immunity even if the same transgene is modified.^360^ C57BL/6 and BALB/c mice have different immune responses, the former being biased toward the Th1 type and the latter toward the Th2 type. BALB/c mice expressing hACE2 may allow modeling the lung pathology and late sequelae of COVID-19.^327^ Wild-type hamsters are permissive for SARS-CoV-2 infection, but may benefit from enhanced susceptibility if the hACE2 gene is introduced.^361^ Similarly, wild-type ferrets can be infected with SARS-CoV-2, and if there native Ace2 gene is humanized, more severe symptoms may be generated. However, it is much more challenging and expensive to genetically modify hamster or ferrets than mouse, since mice benefit from a vast array of mature technology. Nonetheless, some research teams have taken the first step forward in genetically modifying these alternative animal modes.^295,361^

## Reserving animal models in advance, a lesson from the COVID-19 pandemic

Animal production and breeding takes a long time, and the construction of novel genetically modified animal models is even more time consuming. By contrast, the outbreak of an emerging infectious disease is urgent and unpredictable. It is therefore very necessary to maintain a population of naturally susceptible animal models, such as NHPs, ferrets and Syrian hamsters in advance, and to establish genetically modified mouse models for those pathogens lacking susceptible animal models. According to the characteristics of the pathogens and its receptor, genetically modified animal models may be developed by introducing host receptors or co-receptor into mice, resulting in susceptible mouse models. In addition, mice deficient in T, B or NK cell function,^331^ or lacking the interferon (IFN) receptor^362^ are permissive to infection by multiple pathogens. Accordingly, knocking out the host *rag* 2 gene,^363–365^ or IFN receptor gene may result in broad-spectrum susceptible animal models.^362,366,367^

In view of the rapid spread and great harm of respiratory pathogens, more attention should be paid to the construction and reserve of animal models for respiratory pathogens, such as coronaviruses, respiratory syncytial viruses, or enteroviruses that can be transmitted by respiratory routes, such as EV-D68.^368^

**Fig. 2:**
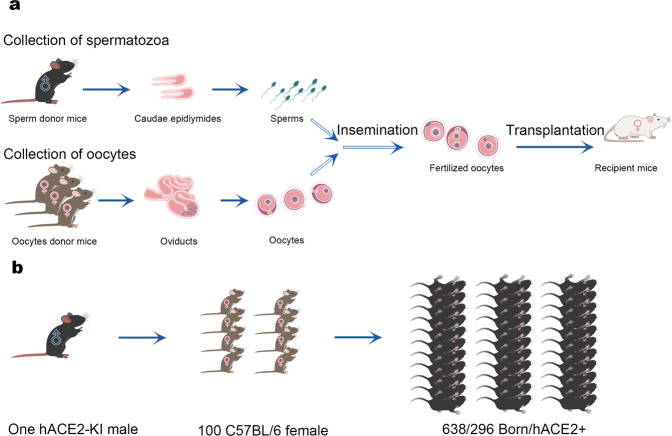
Rapid breeding of hACE2-KI model mice using in vitro fertilization (IVF) technology. **a** The main steps of IVF, including a collection of mouse sperm and oocytes, insemination and implantation of fertilized oocytes into recipient foster mice. **b** A single humanized hACE2-KI mouse and 100 C57BL/6 wild-type mice could produce 638 offspring by IVF technology in a single round, among which 296 hACE2 positive mice were screened by PCR. This rapid breeding technique greatly accelerated the production of humanized hACE2-KI mice

To maintain a stable population of genetically modified mouse models requires large amounts of resources and labor, while cryo-preservation of embryos and sperm costs much less. It is a big challenge to produce thousands of mice for experiments in one or two months when starting with a handful of mice. In vitro fertilization (IVF) technology^369–371^ may achieve this goal.^372^ In one example, sperm from 1 or 2 male hACE2-KI mice^114^ was collected, and hundreds of wild-type C57BL/6 mice were super-ovulated to collect oocytes. Fertilized oocytes were transplanted into recipient mice, and hACE2 positive mice were screened among the offspring (Fig. 2). Over 1000 hACE2 mice were successfully produced in two months in our laboratory (Table 8) in the first half year of 2020, when hACE2 transgenic mice were in great and urgent demand. Such a rapid breeding system was a crucial tool in the initial phase of the COVID-19 pandemic.

**Table 8:** Rapid breeding of hACE2-KI humanized mouse by in vitro fertilization (IVF) technology^a^

No. of times	Sperm donor mice	Oocytes donors	Total oocytes	Average oocytes per mouse	Total born	hACE2 positive
No. 01	1	100	3978	39.78	638	296
No. 02	1	100	3062	30.62	697	325
No. 03	1	120	4698	39.15	1085	474
No. 04	1	120	2926	24.38	416	191
No. 05	2	120	3766	31.38	774	359
No. 06	1	120	3622	30.18	782	350
Total	7	680	22,052	–	4392	1995

^a^Seven humanized hACE2-KI^114^ mice and 680 C57BL/6 wild-type mice could produce 4392 offspring by IVF technology for two months, and 1995 hACE2 positive mice were screened by PCR amplification

## Conclusions

Multiple kinds of COVID-19 disease models have been developed to date, using a wide range of animal models such as NHPs, genetically modified mouse, AAV- or Ad5 transduced mice, as well as wild-type mice infected with adapted strains. In addition, Syrian hamsters, ferrets, as well as livestock or poultry,^24^ including pigs, cats,^306^ dogs, sheep,^305^ chickens and ducks^24^ have also been used as models for SARS-CoV-2 infection. These existing models play crucial roles in the development of vaccines and therapeutics. Nevertheless, outstanding deficiencies have emerged, especially when facing novel requirements. Facing the new phase of COVID-19 pandemic, the scientific community should focus on establishing multi-gene genetically modified animal models and models of VOC infection.^373^ Compared with other species, mice are more easily genetically modified, making it easier to establish complex multi-gene models, and there is an abundant immunological resource. It therefore stands to reason that mouse models will become state-of-the-art tools for replicating complex clinical features and disease progressions of COVID-19.^327^ Finally, there is an urgent need to reserve animal resources and to prepare well for novel emerging infectious diseases in the future, particularly those caused by respiratory pathogens.
